# BjussuLAAO-II induces cytotoxicity and alters DNA methylation of cell-cycle genes in monocultured/co-cultured HepG2 cells

**DOI:** 10.1590/1678-9199-JVATITD-1476-18

**Published:** 2019-03-11

**Authors:** Ana Rita Thomazela Machado, Alexandre Ferro Aissa, Diego Luis Ribeiro, Rui Seabra Ferreira, Suely Vilela Sampaio, Lusânia Maria Greggi Antunes

**Affiliations:** 1 Department of Clinical Analysis, Toxicology and Food Sciences, School of Pharmaceutical Sciences of Ribeirão Preto, University of São Paulo - USP, Ribeirão Preto, SP, Brazil.; 2 Department of Genetics, Ribeirão Preto Medical School, University of São Paulo - USP, Ribeirão Preto, SP, Brazil.; 3 Center for the Study of Venoms and Venomous Animals (CEVAP), São Paulo State University - UNESP, Botucatu, SP, Brazil.

**Keywords:** snake venom, epigenetics, GADD45A, CCND1, CDKN1A

## Abstract

**Background:**

The use of animal venoms and their toxins as material sources for biotechnological applications has received much attention from the pharmaceutical industry. L-amino acid oxidases from snake venoms (SV-LAAOs) have demonstrated innumerous biological effects and pharmacological potential against different cancer types. Hepatocellular carcinoma has increased worldwide, and the aberrant DNA methylation of liver cells is a common mechanism to promote hepatic tumorigenesis. Moreover, tumor microenvironment plays a major role in neoplastic transformation. To elucidate the molecular mechanisms responsible for the cytotoxic effects of SV-LAAO in human cancer cells, this study aimed to evaluate the cytotoxicity and the alterations in DNA methylation profiler in the promoter regions of cell-cycle genes induced by BjussuLAAO-II, an LAAO from *Bothrops jaracussu* venom, in human hepatocellular carcinoma (HepG2) cells in monoculture and co-culture with endothelial (HUVEC) cells.

**Methods:**

BjussuLAAO-II concentrations were 0.25, 0.50, 1.00 and 5.00 μg/mL. Cell viability was assessed by MTT assay and DNA methylation of the promoter regions of 22 cell-cycle genes by EpiTect Methyl II PCR array.

**Results:**

BjussuLAAO-II decreased the cell viability of HepG2 cells in monoculture at all concentrations tested. In co-culture, 1.00 and 5.00 μg/mL induced cytotoxicity (*p* < 0.05). BjussuLAAO-II increased the methylation of *CCND1* and decreased the methylation of *CDKN1A* in monoculture and *GADD45A* in both cell-culture models (*p* < 0.05).

**Conclusion:**

Data showed BjussuLAAO-II induced cytotoxicity and altered DNA methylation of the promoter regions of cell-cycle genes in HepG2 cells in monoculture and co-culture models. We suggested the analysis of DNA methylation profile of *GADD45A* as a potential biomarker of the cell cycle effects of BjussuLAAO-II in cancer cells. The tumor microenvironment should be considered to comprise part of biotechnological strategies during the development of snake-toxin-based novel drugs.

## Background

The use of animal venoms and their toxins as natural sources of material for biotechnological application has received much attention from the pharmaceutical industry and experts in the field of applied research. Since the development of captopril, the first drug derived from a bradykinin-potentiating peptide from *Bothrops jararaca*, to the disintegrins that have displayed potent activity against certain types of cancers, the components of snake venom (SV) have shown great potential for the development of new candidate drugs [1-3]. SV is probably the most complex venom and consists of metalloproteinases, disintegrins, phospholipase A2, lectins, and L-amino acid oxidases (LAAOs) [4].

LAAOs constitute 1-9% of the total venom protein and are responsible for the yellowish coloration of the venom [5]. Many studies of biological characterization of L-amino acid oxidase isolated from SVs (SV-LAAOs) have already demonstrated innumerous biological effects and pharmacological potential of these proteins [6, 7]. In this sense, SV-LAAOs could be used for the development of new and more effective drugs against different types of cancer [8]. 

Cancer is one of the leading causes of death in the world, and there is an urgent need to find better treatments [9]. Hepatocellular carcinoma (HCC) is the most common type of primary cancer of the liver, ranked as the fifth most frequent cancer and the third greatest cause of cancer mortality in the world [10]. HCC is often diagnosed late in individuals with severe hepatic impairment, thus limiting the options for chemotherapy and adjuvant therapies. Also, the lack of early detection markers and drug resistance may contribute to the high mortality rate in HCC [10]. Moreover, increasing evidence suggests that the development of HCC is the result of a multi-step process involving many genetic and epigenetic abnormalities that result in abnormal gene expression [10, 11]. 

HCC tumors display distinct DNA methylation signatures associated with risk factors, tumor stage, degree of differentiation, and survival after antineoplastic therapy [11]. In the HCC genome, hypomethylation affects the structural-nuclear function by promoting chromosomal and genomic instability, whereas hypermethylation is often associated with the silencing of tumor-suppressor genes. Together, these processes determine hyperactivation of progressive steps in hepatocarcinogenesis [12].

Aberrant DNA methylation of Cytosine-phosphate-Guanine (CpG) islands is thought to be one of the most common epigenetic change in cancer, usually resulting in impairment of gene expression. In the mammalian genome, DNA methylation occurs by the covalent addition of a methyl (-CH3) group to cytosine residues in CpG dinucleotides. Nearly 70% of the gene promoters annotated in the human genome are characterized by high CpG content. Methylation of DNA in CpG islands located in gene promoter regions is associated with differential gene expression. The altered epigenetic state may lead to deregulation of cellular processes, such as proliferation, transformation, and anti-apoptotic mechanisms, which promote tumorigenesis [11, 13]. DNA methylation can mediate gene silencing directly by inhibiting the binding of methylation-dependent transcriptional activators or indirectly by altering the affinity of the proteins involved in chromatin remodeling. In cancer cells, the loss of DNA methylation mainly affects the repetitive genomic elements and gene bodies, whereas hypermethylation occurs mainly in the promoters of tumor suppressor genes [10]. 

Recent studies have reported that the tumor microenvironment plays a major role in neoplastic transformation, whereas the presence of endothelial cells also correlates with induction of chemosensitization and chemoresistance [14-16]. In order to better understand the molecular mechanisms responsible for the cytotoxic effects of SV-LAAO on human cancer cells *in vitro*, the purpose of this study was to evaluate the cytotoxicity and the alterations in DNA methylation profile of cell cycle genes induced by BjussuLAAO-II, an LAAO isolated from *Bothrops jararacussu* snake venom, in human hepatocellular carcinoma (HepG2) cells in monoculture and in co-culture with an endothelial cell line (HUVEC).

## Methods

### Toxin

BjussuLAAO-II was isolated from *Bothrops jararacussu* snake venom according to the procedure described by Carone et al. [17]. The toxin is an acidic enzyme that exhibits high enzymatic activity (4,884.53 U/mg/min), has isoelectric point of 3.9 and molecular mass of 60.36 kDa, and represents 0.3% of the venom proteins. Before performing the biological assays, LAAO enzymatic activity was determined by a spectrophotometric assay using L-leucine as a substrate [18]. The isolated and purified protein was stored at 4°C. The vehicle employed to dilute the protein was phosphate buffered saline (PBS, pH 7.4).

### Cell lines and culture conditions

Human hepatocarcinoma cells (HepG2 - catalog #HB8065) and human umbilical-vein endothelial cells (HUVEC - catalog #CRL-1730) were obtained from the American Type Culture Collection (ATCC, Manassas, Virginia, USA). The cells were maintained in RPMI 1640 medium supplemented with 10% FBS, 1% antibiotic-antimycotic solution (5 mg/mL penicillin, 5 mg/mL streptomycin, and 10 mg/mL neomycin), and 0.024% (w/v) NaHCO_3_, in a CO_2_ incubator with 5% atmosphere, at 37 °C and relative humidity of 96%. The media were changed every 2-3 days; when the cultures had reached confluency, the cells were washed twice in PBS, detached with Trypsin/EDTA (0.25%), centrifuged at 174 x *g* for 5 min and sub-cultured. All the experiments were conducted between the third and the eighth cell passage and they were cultured as reported by Bal-Price and Coecke [19].

### Co-culture system

Thincert™ (Greiner Bio-one, Kremsmünster, Austria) cell-culture inserts with 0.4 μm porous polycarbonate membranes in 6-well plates were used in cellular co-culture systems. HepG2 cells (2×10^5^ cells/well) were grown adhering to the bottom of the well whereas HUVEC cells (1×10^4^ cells/well) were grown in the upper compartment [20-23]. The Millicell ERS^®^ volt-ohm meter (Merck-Millipore, Burlington, Massachusetts, USA) was employed to monitor electrical resistivity of HUVEC cells. The inserts whose transepithelial electrical resistance was greater than or equal to 750 Ω/cm^2^ were considered confluent; when this value was reached, HepG2 cells were seeded underneath the well in co-culture plates. Experiments in co-culture systems followed the same protocols described for monoculture systems.

### MTT assay

Cell viability was determined using the MTT assay, as reported by Mosmann [24]. In monoculture systems, HepG2 and HUVEC (1×10^4^ cells/well) were seeded in 96-well plates. In co-culture systems, 6-well plates were used and HepG2 were seeded in the lower (4×10^5^ cells/well) and HUVEC (1×10^4^ cells/well) was placed in upper compartments. In both systems, cells were incubated for 24 h and treated with BjussuLAAO-II (0.25; 0.50; 1.00 and 5.00 μg/mL), PBS (negative control) or methyl methanesulfonate (MMS; CAS: 66-27-3; positive control) for 72 h. The supernatant was removed, and 0.2 mL or 3.0 mL of MTT solution (5 mg/mL) were added to the wells in mono- and co-culture systems, respectively. After 3 h of incubation, the supernatant was replaced by equivalent volumes of DMSO (Sigma Aldrich, St. Louis, Missouri, USA) and absorbance was recorded in a spectrophotometer (Biotek Elx800 - Winooski, VT, USA) set at 570 nm. Absorbance values of the negative control were defined as constituting 100% cell viability, and the results were expressed as a percentage (%) of viable cells.

### EpiTect methyl qPCR array analysis

HepG2 cells in mono- and co-culture were cultivated as described in the MTT assay. The methylation of the promoter region of 22 cell-cycle genes was analyzed using EpiTect Methyl II PCR Array Human Cell Cycle Signature PCR Array (EAHS-201ZC-2, Qiagen, Hilden, Germany) following the manufacturer's protocol. Genes are related to the G1 phase (*CCND1*,*CCNE1*,*CDK4*, *CDKN1*B), S Phase and Replication (*MCM2*,*MCM4*), G2 Phase & G2/M Transition (*CCNB1*,*CDK5RAP1*,*CKS1B*), M Phase (*CCNF*,*MRE11A*,*RAD51*), Cell-Cycle Checkpoint & Cell-Cycle Arrest *(ATM, BRCA1, BRCA2, CDK2, CDKN1A, CDKN1B, CHEK1, GADD45A, RAD9A, TP53*) and Regulation of Cell Cycle (*ATM, BRCA1, BRCA2, CCNB1, CCND1, CCNE1, CCNF, CDK2, CDK4, CDKN1A, CDKN1B (p27KIP1), CKS1B, GADD45A, RAD9A, RBL1, TP53*). This assay is based on the digestion of unmethylated and methylated DNA, using methylation-sensitive and methylation-dependent restriction enzymes. Genomic DNA from HepG2 treated for 72 h with 0.25 µg/mL of BjussuLAAO-II was isolated using the DNeasy Blood and Tissue Kit (Cat #69504, Qiagen, Hilden, Germany) according to the manufacturer’s protocol. The genomic DNA was quantified by spectrophotometry (NanoDrop 2000C; Thermo Scientific, San Jose, CA, USA) with all samples showing a A260/A280 ratio higher than 1.8. The digestion was performed employing the EpiTect Methyl II DNA Restriction Kit (Qiagen, Hilden, Germany). A reaction mix without enzymes was prepared from 1 μg genomic DNA, 26 μL of 5× Restriction Digestion Buffer, and RNase-DNase-free water to make the final volume 120 μL. Four digestion reactions - Mo (no enzyme - mock digestion), Ms (methylation sensitive), Md (methylation-dependent), and Msd (double) were set up. The samples were incubated at 37°C for 6 h. Then quantitative PCR (qPCR) was performed using the RT2 SYBR Green ROX qPCR Master Mix (Qiagen, Hilden, Germany) according to the manufacturer’s recommendations in a Step One Plus Real-Time PCR System (Applied Biosystems, Foster City, CA, USA). Threshold cycle (Ct) values were utilized to calculate the percentages of methylated (M) and unmethylated (UM) DNA, using a quantization algorithm provided by the manufacturer to normalize the amount of DNA in each digestion against the total amount of input DNA in the mock digestion, using the Excel macro-spreadsheet supplied by the manufacturer (Qiagen, Hilden, Germany) [25-27]. 

### Statistical analysis

All the results were expressed as the mean ± standard deviation (SD) of triplicates (n=3). Kolmogorov-Smirnov test was employed to test the normality, and experimental data were analyzed using one-way analysis of variance (ANOVA) followed by the Tukey’s test in MTT assay and Dunnett’s test in DNA methylation assay. Data were statistically analyzed using the software GraphPad Prism 5.00 for Windows (GraphPad Software, La Jolla, CA, USA) and statistical significance was considered at *p* < 0.05.

## Results

### BjussuLAAO-II is cytotoxic towards HepG2 and HUVEC cells

**Figure 1:**
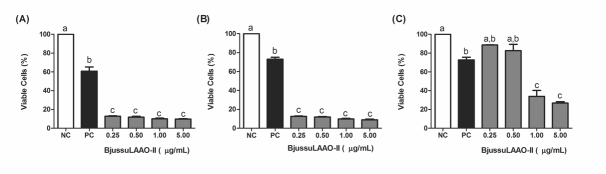
Cell viability of HepG2 and HUVEC cells treated with BjussuLAAO-II for 72 h. **A**: HepG2 cells in monoculture. **B**: HUVEC cells in monoculture. **C**: HepG2 cells in co-culture. NC: Negative Control (PBS, pH 7.4). PC: Positive Control (300 µM MMS). Percentage (%) of cell viability, assessed by the MTT assay, was calculated for the NC and expressed as mean ± SD (n = 3). Bars not sharing the same letter are significantly different from each other (*p* < 0.05; ANOVA followed by the Tukey test).

At all concentrations tested (in the range from 0.25 to 5.00 µg/mL), BjussuLAAO-II decreased the MTT reduction capacity in HepG2 (Fig. 1A) and HUVEC (Fig. 1B) cells in monocultures. In HepG2 co-culture, only the 1.00 and 5.00 µg/mL concentrations significantly decreased the cell viability (Fig. 1C) when compared to the negative control. MMS (300 µM), used as positive control, was effective at decreasing cell viability in HepG2 and HUVEC in both monoculture and in co-culture.

### BjussuLAAO-II altered DNA methylation in the promoter region of cell-cycle genes

**Figure 2:**
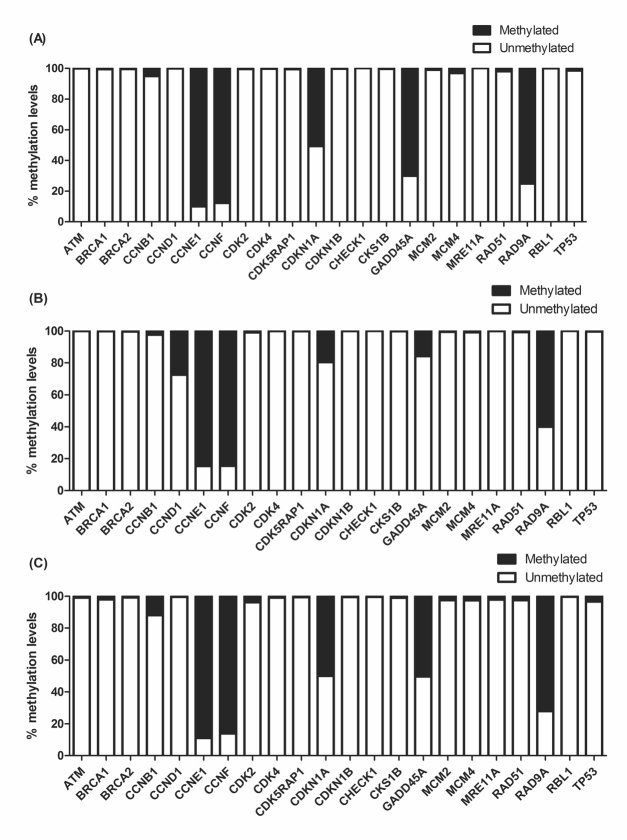
DNA methylation profile in promoter region of cell cycle genes after 72h of BjussuLAAO-II treatment. **A**bold>: HepG2 cells in monoculture treated with PBS (pH 7.4), used as negative control. **B**: HepG2 cells in monoculture treated with BjussuLAAO-II (0.25 µg/Ml). **C**: HepG2 cells in co-culture treated with BjussuLAAO-II (0.25 µg/mL).

**Figure 3:**
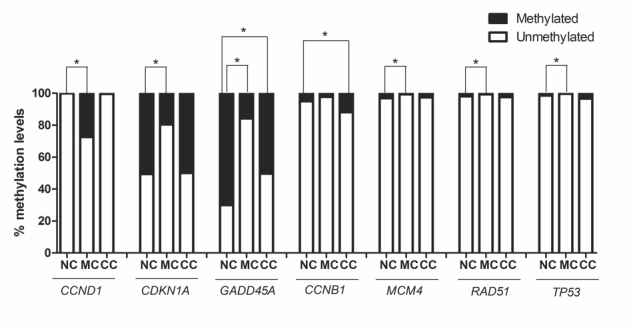
Genes with significant changes in DNA methylation in promoter region after 72h of BjussuLAAO-II treatment. NC: HepG2 cells in monoculture treated with PBS (pH 7.4), used as negative control. MC: HepG2 cells in monoculture treated with BjussuLAAO-II (0.25 µg/Ml). CC: HepG2 cells in co-culture treated with BjussuLAAO-II (0.25 µg/mL). *Values with significant difference (*p* < 0.05; ANOVA followed by Dunnett’s test) compared to NC.

Since 0.25 µg/mL of BjussuLAAO-II was not cytotoxic in co-culture, this concentration was selected to assess the effect of the toxin on the DNA methylation of the promoter region of 22 cell-cycle genes in HepG2 cells in monoculture and co-culture (Fig. 2 and Fig. 3). As can be seen in Fig. 3, after 72 h of exposure, of the total of 22 cell-cycle genes, six were statistically altered in monoculture treatment and two genes were altered in co-culture when compared to the respective negative control. As to DNA methylation in monoculture, *CCND1* showed an increase while *CDKN1A, GADD45A*, *MCM4, RAD51* and *TP53* presented a decrease. In co-culture, BjussuLAAO-II decreased the DNA methylation of *GADD45A* and increased the DNA methylation of *CCNB1* (Fig. 3). To select the genes with a more biologically relevant alteration, a methylation change threshold of >20% was applied in non-treated cells [28]. *CCND1* showed a methylation decrease from 100% in negative control to 72% in BjussuLAAO-II-treated cells in monoculture. *CDKN1* produced methylation of 49% in negative control and 80% in monoculture, i.e., a 31% increase of DNA methylation induced by BjussuLAAO-II. *GADD45A* was the gene most influenced by the BjussuLAAO-II since in monoculture the DNA methylation of its promoter region increased from 30% in negative control to 80% in monoculture and 50% in co-culture. 

## Discussion

Biotechnological strategies are needed for the development of new drug candidates to improve the treatment of cancer. The search for antineoplastic drugs obtained from natural biological resources is global. Venoms and toxins are rich in several bioactive substances, which makes them an excellent source for the discovery of new drugs. Thus, it is important to assess the anticancer effects of animal toxins on human cancer cells to decide the best candidates for potential clinical use. *In vivo* and *in vitro* studies have demonstrated that animal toxins, either isolated or in raw venom, have inhibitory effects against cancer cells [29, 30]. Many SV-LAAOs isolated from different snake species have been described as capable of inducing cell death in diverse cell lines by reducing the cell viability measured by the MTT assay. Previous studies have shown that cytotoxicity is an important function of these enzymes [31, 32].

OH-LAAO, isolated from the *Ophiophagus hannah* snake, has demonstrated antiproliferative activity in human breast cancer cells (MCF-7) and in human lung adenocarcinoma cells (A549) after 72h of treatment [33]. This antiproliferative effect is highly selective, with IC_50_ values being 3- and 4-fold higher in non-tumor cells of human mammary gland (184B5) and bronchial epithelium (NL20), respectively [33]. BjussuLAAO-II after 72h of treatment also reduced cell viability in HepG2. Despite reports that claimed selective cytotoxicity of SV-LAAO in cancer cell lines, LAAOs from some species have exhibited cytotoxic effects on non-tumor cells as well [5]. Similar results are reported in the present study from BjussuLAAO-II since the toxin reduced the viability of the non-tumor HUVEC in the MTT assay. In a previous study, BjussuLAAO-II was tested against human colorectal adenocarcinoma cells (Caco-2) in a 24h treatment and also demonstrated the reduction of cell viability [34].

It has been proposed that the specific effects of SV-LAAOS on biological systems are due to the generation of hydrogen peroxide [6, 31, 35]. However, the molecular mechanisms by which SV-LAAOs induce cytotoxicity and apoptosis in cancer cells are not fully understood. 

There are few reported studies in the literature on the DNA methylation changes or histone modification in response to snake venoms or snake toxins *in vitro*. Wu et al. [36] found that melittin, a major component of bee venom, can inhibit human hepatocarcinoma (SMMC-7721) cell proliferation due to delay in cell-cycle progression. The results showed that melittin decreases the methylation of *PTCH1*, which is important for tumorigenesis, resulting in the increased expression of its protein *in vitro*. In order to provide information about the involvement of epigenetic regulation in a rat model of bee venom-induced inflammatory pain, Yang et al. [37] investigated selective class I histone deacetylase inhibitors (HDACIs) in rats inflamed by subcutaneous injection of whole venom from *Apis millifera*. Changes in histone deacetylase 1 (*HDAC1*) and 2 (*HDAC2*) expression patterns were observed in intact rat lumbar spinal cord after bee venom injection and treatment with *HDCA* inhibitors. 

DNA methylation of CpG islands is the best known important epigenetic mechanism in cancer progression. Aberrant DNA methylation of CpG islands is thought to be one of the most common changes in the silencing of cell-cycle genes in various tumor types [38]. Gene regulation is controlled by different mechanisms, such as epigenetic processes, that influence several essential pathways and stages of carcinogenesis including tumor initiation and progression. Deregulation of the cell cycle is a common characteristic of human cancer while cell-cycle regulatory proteins are critical determinants of malignancy progression. Genes controlling G1, S, G2 and M phases of the cell cycle - including cyclin-dependent kinases (CDKs), cyclins activating CDKs and cyclin-dependent kinase inhibitors (CDKIs) - are crucial for the control of mammalian cell proliferation [38, 39]. 

The genes most affected by BjussuLAAO-II, with an alteration higher than 20% as compared with non-treated cells, were *CDKN1A* (Cyclin-Dependent Kinase Inhibitor 1A), *CCND1* (Cyclin D1), and *GADD45A* (Growth Arrest and DNA-Damage-Inducible 45 Alpha). *CDKN1A* controls the proliferation, differentiation and tumorigenesis of many cancer cell types and plays a key role in the p53-mediated cell cycle arrest [40]. Generally, *CDKN1A* has been observed hypermethylated and down-regulated in HCC tissues, which might promote cell-cycle progression with consequent uncontrolled proliferation of liver cells with pre-cancerous lesions thereby contributing to oncogenesis [10, 41, 42]. BjussuLAAO-II decreased the methylation of the promoter region of this gene, which can return to the normal epigenetic status, increasing gene expression and stopping the cell cycle. In fact, a decrease was observed in cell viability as measured by the MTT assay in HepG2 cells. Similar effects were reported by Obata et al. [40], who found that hypomethylation of the *CDKN1A* promoter region resulted in cell-cycle arrest in intestinal regulatory T cells *in vitro*.

*CCND1* is a key cell-cycle regulator and one of the most important oncogenes, being overexpressed in several human cancers. CCND1 forms a complex with CDK4 to control cell-cycle progression through the G1 phase and to promote the cell-cycle transition into the S phase by activation of the CCNE1/CDK2 complex [43, 44]. Overexpression of *CCND1* disrupts the normal cell cycle, possibly promoting the development and progression of cancer [43]. However, it does not exert tumorigenic activity by itself but rather cooperates with other altered oncogenes and tumor suppressors to induce and govern neoplastic transformation [44]. Lin et al. [45] demonstrated that Protease Serine 3 (PRSS3) exerts tumor-suppressive functions in human HCC by inducing G1/S cell-cycle arrest and suppressing the complexes of CCND1/CDK4. The results reported herein suggest that BjussuLAAO-II may also induce cell-cycle arrest by increasing the methylation of *CCND1*. 

*GADD45A* is a tumor suppressor gene that has cell-type-specific roles in cellular stress, coordinating DNA repair and demethylation, cell-cycle arrest and pro-apoptotic or pro-survival responses [46]. An upregulation of *GADD45A* has been shown to protect against DNA damage and uncontrolled cellular proliferation [47]. *GADD45A* family members are multi-faceted nuclear factors implicated in active DNA demethylation, apart from maintenance of genomic stability, DNA repair and suppression of cell growth in vertebrates [48]. The hypomethylation of the *GADD45A* gene caused by BjussuLAAO-II may increase its expression leading to the demethylation of tumor suppressor genes, increasing their expression and consequently, decreasing cell proliferation in monoculture. There was also a decrease in DNA methylation of the *GADD45A* in HepG2 cells in co-culture, evidencing that BjussuLAAO-II is able to alter the methylation pattern of this gene even in the tumor microenvironment. Serum *GADD45A* methylation was previously indicated as a useful biomarker to distinguish benign versus malignant prostate cancer [49]. Moreover, we suggested that *GADD45A* may serve as a suitable biomarker of the effects of BjussuLAAO-II on DNA methylation, since this gene is indicated to distinguish between benign versus malignant prostate disease in patients.

The tumor microenvironment plays an essential role in proliferation, migration, survival and drug resistance in human tumors [50] and in cell culture [51]. For example, hepatocyte growth factor, one of the important growth factors in the tumor microenvironment, upregulated the expression of DNA Methyltransferase 1 (*DNMT1*) in HepG2 and non-cancer human liver cells (HL-7702) cells and the overexpression of *DNMT1* in HCC patients correlated with the malignant potential and poor prognosis [52]. For this reason, the use of co-cultures, such as HepG2 co-cultured with endothelial cells, is an important tool to understand the tumor microenvironment in hepatocarcinoma development [16]. As can be observed both in the MTT assay and in the DNA methylation in the promoter region of cell cycle genes, the tumor microenvironment plays an important role in the cellular response to BjussuLAAO-II treatment. 

## Conclusion

Our findings may provide new insights into the mechanisms by which toxin snake venom is cytotoxic against hepatocellular carcinoma cells. Specifically, BjussuLAAO-II may exert its cytotoxicity in HepG2 cells by decreasing the methylation of tumor-suppressor genes including *CDKN1A* and *GADD45A* and increasing the methylation of oncogenes such as *CCND1*. We suggest the analysis of the DNA methylation profile of *GADD45A* as a potential biomarker of the cell-cycle effects of BjussuLAAO-II in cancer cells. Furthermore, cell viability and DNA methylation changes were dependent on the cell-culture model. For this reason, we proposed that the role of the tumor microenvironment should be considered in biotechnological studies investigating novel chemotherapeutic drugs rather than only *in vitro* monoculture assays.

## Abbreviations

BjussuLAAO-II: LAAO from *Bothrops jararacussu* venom; *CCND1*: Cyclin D1; *CDKN1A*: Cyclin-Dependent Kinase Inhibitor 1A; *CpG*: Cytosine-phosphate-Guanine island; DNMT1: DNA Methyltransferase-1; *GADD45A*: Growth Arrest And DNA-Damage-Inducible 45 Alpha; HCC: Hepatocellular carcinoma; HepG2: Hepatocarcinoma cells; HDAC: Histone deacetylase inhibitors; HUVEC: Human umbilical vein endothelial cells; LAAO: L-amino acid oxidase; MMS: Methyl methanesulfonate; MTT: 3-(4,5-dimethylthiazol-2-yl)-2,5-diphenyltetrazolium bromide; Mo: No enzyme digestion; Ms: Methylation-sensitive enzyme digestion; Md: Methylation-dependent enzyme digestion; Msd: Double-enzyme digestion; PBS: Phosphate Buffered Saline; SV: Snake Venom; SV-LAAO: LAAO from snake venom.
